# The *SlDLK2* receptor, involved in the control of arbuscular mycorrhizal symbiosis, regulates hormonal balance in roots

**DOI:** 10.3389/fmicb.2024.1472449

**Published:** 2024-12-11

**Authors:** Martín Ramos-Alvelo, Nuria Molinero-Rosales, María Isabel Tamayo-Navarrete, Sanja Ćavar Zeljković, Petr Tarkowski, José Manuel García-Garrido, Tania Ho-Plágaro

**Affiliations:** ^1^Department of Soil and Plant Microbiology, Estación Experimental del Zaidín (EEZ), CSIC, Granada, Spain; ^2^Czech Advanced Technology and Research Institute, Palacky University, Olomouc, Czechia; ^3^Centre of the Region Haná for Biotechnological and Agricultural Research, Department of Genetic Resources for Vegetables, Medicinal and Special Plants, Crop Research Institute, Olomouc, Czechia

**Keywords:** arbuscular mycorrhiza, plant hormones, *DLK2*, transcriptomics, tomato

## Abstract

Arbuscular mycorrhiza (AM) represents a symbiotic mutualistic association between most land plants and *Glomeromycota* fungi. AM fungi develops specialized intraradical and highly branched structures, called arbuscules, where bidirectional exchange of nutrients between plant and fungi partners occurs, improving plant growth and fitness. Transcriptional reprogramming and hormonal regulation are necessary for the formation of the arbuscules. *SlDLK2*, a member of the third clade from the DWARF14 family of *α*, *β*-hydrolases closely related to the strigolactone receptor D14, is a negative regulator of arbuscule branching in tomato, but the underlying mechanisms are unknown. We explored the possible role of *SlDLK2* on the regulation of hormonal balance. RNA-seq analysis was performed on roots from composite tomato plants overexpressing *SlDLK2* and in control plants transformed with the empty vector. Analysis of transcriptomic data predicted that significantly repressed genes were enriched for genes related to hormone biosynthesis pathways, with a special relevance of carotenoid/apocarotenoid biosynthesis genes. Stable transgenic *SlDLK2* overexpressing (OE) tomato lines were obtained, and hormone contents were analyzed in their roots and leaves. Interesting significant hormonal changes were found in roots of *SlDLK2* OE lines with respect to the control lines, with a strong decrease on jasmonic acid and ABA. In addition, *SlDLK2* OE roots showed a slight reduction in auxin contents and in one of the major strigolactones in tomato, solanacol. Overall, our results suggest that the negative regulation of AM symbiosis by *SlDLK2* is associated with the repression of genes involved in the biosynthesis of AM-promoting hormones.

## 1 Introduction

Arbuscular mycorrhiza (AM) represents a symbiotic mutualistic association between most land plants and fungi from the *Glomeromycota*. The interaction benefits plant and fungi with the exchange of nutrients between the two partners. Plants in association with AM fungi improve their growth and fitness, and AM fungi receive plant carbohydrates and lipids essentials for their development (Shi et al., 2023).

Functional AM development requires fundamental reprogramming of root cells, to allow the formation of symbiotic structures required for nutrient exchange. Several stages in the establishment of AM have been identified, including the exchange of diffusible signals for mutual recognition, induction of AM-related genes in the host for cellular rearrangement that allows accommodation of the AM fungus, and creation of the arbuscule, the symbiotic structure which provides an appropriate interface for the exchange of nutrients (Choi et al., 2018).

The interaction is highly regulated by both partners, namely, plant and AM fungi, at the cellular, molecular, and genetic levels. Host plant cells regulate the development and functioning of the mutualistic association by a complex transcriptional reprogramming that includes, among others, hormone-related genes (mainly strigolactones and gibberellins), common symbiotic signaling pathway (CSSP) genes, transcription factors, and genes for transport, metabolism, and cellular processes required for functional AM symbiosis (Ho-Plágaro and García-Garrido, 2022). Particularly important are the extensive transcriptional changes that are induced during arbuscular formation, and a precise spatiotemporal regulation of gene expression is essential for proper arbuscule development. Therefore, the identification of the mechanisms mediating these gene expression changes is crucial to understand how arbuscule formation and function are regulated (Pimprikar and Gutjahr, 2018).

Several studies have highlighted the potential role of apocarotenoids and related compounds in regulating the arbuscular mycorrhizal symbiosis cycle. In earlier research, Ho-Plágaro et al. (2021) recently identified a tomato gene encoding an apocarotenoid-like receptor protein, *DLK2*, which plays a regulatory function in arbuscule formation. *DLK2* proteins form a third clade within the DWARF14 family of *α*, *β*-hydrolases, closely related to the strigolactone receptor D14. The expression of the *DLK2* gene has consistently been used as a marker for strigolactone (SL) and karrikin (KAR) signaling (Waters et al., 2012; Sun et al., 2016). SLs, which are plant hormones derived from carotenoids, were initially identified as soil compounds that trigger the germination of the parasitic plant *Striga lutea* (Cook et al., 1966). They were later discovered to play a crucial role in facilitating the symbiotic relationship between arbuscular mycorrhizal fungi (AMF) and plant roots (Akiyama et al., 2005; Akiyama et al., 2010) and as important regulators of plant development (Gomez-Roldan et al., 2008; Umehara et al., 2008). KARs, a group of butenolide compounds found in smoke, were first identified as stimulants for seed germination in fire-following adapted species. Genetic analysis of KAR signaling revealed an unexpected link to SLs. There is compelling evidence suggesting that KARs act as natural analogs of an unidentified endogenous signal known as the KAI2 ligand (KL). This KAR/KL signaling pathway regulates various plant developmental processes, including germination, photomorphogenesis in seedlings, and root and root hair growth (Waters and Nelson, 2023). In addition, KAR/KL signaling has been shown to influence arbuscular mycorrhizal symbiosis (Gutjahr et al., 2015).

Tomato *DLK2* (*SlDLK2*) is a new component of the complex plant-mediated mechanism regulating the life cycle of arbuscules in AM symbiosis. Interestingly, *SlDLK2* interacts with DELLA, a protein that regulates arbuscule formation/degradation in AM roots (Ho-Plágaro et al., 2021). The DELLA-gibberellin module plays a central role in regulating arbuscule formation (Floss et al., 2013; Martin-Rodriguez et al., 2016). In a complex with DELLA proteins, CYCLOPS regulates the expression of *RAM1* (Pimprikar et al., 2016), which encodes a GRAS-domain transcription factor that constitutes a master regulator for the expression of genes involved in arbuscule development and nutrient exchanges (Rich et al., 2015).

The previous study by Ho-Plágaro et al. (2021) showed that *SlDLK2* ectopic expression downregulates AM-responsive genes, even in the absence of symbiosis, including well-known AM marker genes involved along several stages of arbuscule life cycle. In the present study, we performed an in-depth analysis of changes directed by *SlDLK2* overexpression (OE) in tomato roots based on previous RNA sequencing data. We compared and evaluated in detail differentially expressed genes and the associated gene ontology (GO) terms enriched in tomato roots under two different conditions: ectopic overexpression of *SlDLK2* or AM colonization. Our primary aim was to identify differentially expressed genes involved in the response of tomato roots to AM formation and mediated by *SlDLK2*, and we found a clear overrepresentation of genes involved in different hormone biosynthesis pathways important for AM symbiosis. Further hormone content analyses confirmed that *SlDLK2* has a relevant role in regulating hormonal balance in the roots.

## 2 Materials and methods

### 2.1 RNA sequencing data analysis from previous experiments

For the analysis of transcriptional changes undergoing arbuscular mycorrhization and *SlDLK2* overexpression, raw RNA-seq data obtained from previous experiments were used (NCBI BioProjects PRJNA509606 and PRJNA523214, respectively) (Ho-Plágaro et al., 2021). Data belonged to three root pool samples for each condition: mycorrhized roots inoculated with the AM fungus *Rhizophagus irregularis* and non-inoculated roots (Experiment 1), and *SlDLK2* overexpressing (*SlDLK2* OE) hairy roots and control roots transformed with the empty vector (Experiment 2). RNA-seq sequence processing was performed as detailed in Ho-Plágaro et al. (2021). Gene Ontology enrichment analysis was performed using the PANTHER database (Mi et al., 2021). To identify possible altered hormone-related pathways in response to *SlDLK2* OE on tomato roots, overrepresentation analyses on the significantly *SlDLK2* OE-induced and repressed genes (fold change >2 or < −2, respectively, and *p*-value<0.05) were performed using MetGenMap (Joung et al., 2009). Gene expression heatmaps were generated by z-normalization of log2 count values of selected genes using Heatmapper (Babicki et al., 2016).

### 2.2 RNA sequencing analysis of mycorrhized hairy root plants overexpressing *SlDLK2*

For the analysis of transcriptional changes undergoing *SlDLK2* overexpression in mycorrhizal plants, an experiment with mycorrhizal composite plants overexpressing *SlDLK2* was set up.

The pUBIcGFP-DR: *SlDLK2* vector obtained in Ho-Plágaro et al. (2021) was used for *A. rhizogenes* transformation, and hairy root composite plants were obtained as described in Ho-Plágaro et al. (2018) and Ho-Plágaro et al. (2021). Composite tomato plantlets were inoculated with *Rhizophagus irregularis* and grown as explained in the “Plant growth and AM inoculation” methodological section.

50 days after AM inoculation, root samples from three control plants transformed with the empty vector (31.67 ± 2.62% mycorrhizal colonization) and three *SlDLK2* OE composite plants (19.67 ± 3.72% mycorrhizal colonization) were collected. Total RNA was extracted using the Rneasy Plant Mini Kit (Qiagen). The quality and quantity of total RNA samples were assessed using a NanoDrop 1,000 spectrophotometer (Thermo Scientific), and samples were normalized at the same concentration (6 μg, 300 ng μl^−1^). Later, samples were sent to Sistemas Genómicos SL (Paterna, Valencia, Spain) for cDNA library preparation and sequencing using an Illumina HiSeq1000 machine.

For RNA-seq sequence processing, the TOPHAT v.2.1.0 algorithm (Trapnell et al., 2009) was used to align reads from the RNA-seq experiment to the Tomato Genome Reference Sequence SL3.0 provided by the Sol Genomics consortium at^1^, using the ITAG 3.10 annotation. Then, low-quality reads were removed from the map through Picard Tools^2^, and high-quality reads were selected for assembly and identification through Bayesian inference using the CUFFLINKS v.2.2.1 algorithm proposed by Trapnell et al. (2010). Gene quantification process was performed by the HTSEQ-COUNT 0.6.1p1 tool (Anders et al., 2015). Isoform quantification and differential expression was carried out through the DESEQ2method (Anders et al., 2015). The RNA-seq data have been deposited in the NCBI Short Read Archive (SRA) with accession no. PRJNA509606.

### 2.3 Tomato stable transformation and selection of transgenic lines

Tomato stable transformation was carried out in the laboratory of Tissue culture and plant breeding at the Institute for Plant Molecular and Cellular Biology (IBMCP, Valencia, Spain). The genetic construction in the pK7FWG2 plasmid was introduced into *Agrobacterium tumefaciens* LBA4404, and *Agrobacterium-*mediated transformation of Moneymaker tomato cultivar cotyledons was performed as previously described (Ellul et al., 2003). The empty vector pK7FWG2 was used for the obtention of the control tomato lines.

To induce rooting, the elongated shoots obtained after subculture of the buds were grown in Murashige and Skoog medium (Murashige and Skoog, 1962) supplemented with 0.1 mg l^−1^ indole-3-acetic acid (IAA) and 50 mg l^−1^ kanamycin. T0 plants were grown in soil under standardized greenhouse conditions. After successive self-pollination events, T2 progenies were screened for kanamycin resistance conferred by the NEOMYCIN PHOSPHOTRANSFERASE II (NPTII) marker gene, and azygous (null resistance to kanamycin) and homozygous (100% kanamycin resistance in the progeny plants) lines were identified according to results from this test. Kanamycin test was carried out by sowing seeds on Murashige and Skoog (MS) agar medium supplemented with sucrose (10 g l^−1^) and kanamycin (100 mg l^−1^). PCR analysis on DNA extracted from leaves was performed to corroborate the presence of the kanamycin-resistant *nptII* gene insert. Two azygous control lines transformed with the empty vector (WT-1 and WT-2) and three homozygous independent T2 lines overexpressing *SlDLK2* (OE-1; OE-2 and OE-3) were selected for further studies, and the *DLK2* expression level was analyzed by quantitative real-time polymerase chain reaction (RT-qPCR) using specific primers (Supplementary Table 1). Azygous plants are considered ideal controls because they have been submitted to the entire process of transformation for generating transgenic plants but they have lost the transgene through segregation.

### 2.4 Hormone extraction and analysis

Plant hormone analysis was performed at the Plant Hormones Quantification platform (IBMCP, Valencia, Spain) by Ultra-Performance Liquid Chromatography–Mass Spectrometry (UPLC-MS), using a Thermo Scientific™ Q Exactive™ Hybrid Quadrupole-Orbitrap Mass Spectrometer. In brief, 50 mg of lyophilized leaves or roots material was ground in liquid nitrogen, homogenized in 80% methanol −1% acetic acid containing internal standards, and subjected to gentle agitation for 1 h at 4°C. The resulting extract was maintained at 20°C overnight and then centrifuged, and the supernatant was dried in a vacuum evaporator. The dry residue was suspended in 1% acetic acid, filtered through an Oasis HLB column (Waters Corp., Milford, MA, USA), and subjected to chromatographic separation (Hernandez et al., 2021).

### 2.5 Strigolactone analysis

As strigolactone (SL) production in the roots is promoted under Pi-deficiency conditions (López-Ráez et al., 2008), a phosphate-starvation experiment was set up for strigolactone analysis in tomato root exudates. Plants were grown in a 1:1 mixture of washed vermiculite and sand in 500 mL pots. Initially, plants were watered with 20 mL of complete Long Ashton nutrient solution (Hewitt, 1966) three times a week for 2 weeks. The substrate was then washed with 1 liter of tap water before starting the phosphorus (P) treatments. For the following 2 weeks, plants were watered daily with 25 mL of either standard phosphorus Long Ashton solution for the control treatment (5.2 mM Pi, +P) or phosphorus-free solution for the P-starvation treatment (0 mM Pi, –P) as Pi-deficient culture conditions promote exudation and detection of SLs such as orobanchol, solanacol, and didehydro-orobanchol(s) (López-Ráez et al., 2008; Rial et al., 2019). Subsequently, the substrate was washed with 500 mL of the respective nutrient solution (+P or –P) to remove accumulated compounds. Plants were kept in a growth chamber for 48 h and irrigated to field capacity with the corresponding nutrient solution after 24 h. After this period, fresh root exudates were collected by washing the substrate with 1 liter of tap water, and roots were weighed and stored at −80°C for future analysis. Exudates were vacuum-filtered through glass filters, concentrated, and purified using Telos C18 SPE columns (Telos, Kinesis, UK) and an SPE vacuum manifold (Supelco). SPE columns were first pre-equilibrated with 5 mL of 100% acetone and washed with 5 mL of distilled H_2_O, and then, a liter of each exudate solution was loaded onto the pre-equilibrated columns. Each column was washed with 5 mL of 40% acetone, and the exudates were eluted with 5 mL of 60% acetone and collected in 10 mL amber tubes. Purified root exudates were stored at −80°C until use.

For strigolactone (SL) analysis, a 15 μL aliquot of 25 nM GR24 (internal standard) was added to 150 μL of purified root exudate. The mixture was evaporated to dryness, redissolved in 15 μL of acetonitrile (ACN), and analyzed using Nexera X2 UHPLC coupled with MS-8050. Chromatographic separation was performed on an ACQUITY BEH C18 column with specific gradient elution parameters. Mass spectra were obtained using electrospray ionization in positive mode, and SLs were identified by comparing retention times and MRM transitions with authentic standards. Data processing was performed using LabSolutions 5.72 software.

### 2.6 Plant growth and AM inoculation

*Solanum lycopersicum* seeds were surface-sterilized with a 2.35% sodium hypochlorite solution for 5 min, shaken at room temperature for 1 day in the dark, and germinated on sterilized moistened filter paper at 25°C in the dark for 4 days. Germinated seeds were then placed on vermiculite for 1 week to allow hypocotyl elongation. Each seedling was transferred to a 500 mL pot containing a sterilized mixture of expanded clay, vermiculite, and coconut fiber (2:2:1). For arbuscular mycorrhizal (AM) treatments, plants were inoculated with 200 spores of *Rhizophagus irregularis*. Plants were grown in a growth chamber with a 16-h light/8-h dark cycle at 24°C/20°C and 50% relative humidity. 1 week after planting, and weekly thereafter, pots received 20 mL of modified Long Ashton nutrient solution with 325 μM phosphorus to avoid mycorrhizal inhibition. Non-mycorrhizal plants received the same nutrient solution. Plants were harvested at 57 and 77 days post-inoculation (dpi), with roots washed and rinsed for different measurements. The non-vital trypan blue histochemical staining procedure and the assessment of the intensity of root cortex colonization by AM fungus were performed as described by Ho-Plágaro et al. (2020).

### 2.7 RNA extraction and qPCR analysis

For the quantitative reverse transcription-PCR (RT-qPCR) experiments, representative root samples were collected, frozen in liquid nitrogen, and stored at −80°C until RNA extraction. Approximately 0.2 g of root samples were used to isolate total RNA with the RNeasy Plant Mini Kit (Qiagen), followed by DNase treatment. One microgram of DNase-treated RNA was reverse-transcribed into cDNA using the iScript™ cDNA synthesis kit (BioRad). For the qPCR, a 20 μL reaction mixture was prepared containing 1 μL of diluted cDNA (1:10), 10 μL of 2× SYBR Green Supermix (BioRad), and 200 nM of each primer in a 96-well plate. A negative control without reverse transcription was included to check for DNA contamination. The PCR program included a 3-min incubation at 95°C, followed by 35 cycles of 30 s at 95°C, 30 s at 58–63°C, and 30 s at 72°C, with a melting curve analysis performed after the final cycle. Experiments were conducted on three biological replicates, with each biological replicate having three technical replicates. The threshold cycle (Ct) values were normalized to the geometric mean of Ct values from the housekeeping genes *SlEF-1a* (accession no. X14449) and *SlActin2* (NM_001321306.1). Relative transcription levels were calculated using the 2^−ΔΔCt^ method (Livak and Schmittgen, 2001), and the RT-qPCR data were shown as relative expression compared to a reference treatment (to which a value of 1 was assigned). The genes analyzed and corresponding primers are listed in Supplementary Table 1.

### 2.8 Statistical methods

Comparisons among all means were performed using a one-way analysis of variance (ANOVA) followed by Holm–Sidak’s multiple comparison test. The GraphPad Prism v 8.0.2 (GraphPad Software, Boston, MA, USA) was used to determine statistical significance. Differences at a *p-value of* < 0.05 were considered significant.

## 3 Results

### 3.1 Gene ontology analysis reflects the negative regulatory role of *SlDLK2* on mycorrhization

For Gene Ontology (GO) analyses, significantly induced or repressed genes (fold change >2 or < −2, respectively; *p*-value <0.05) upon *SlDLK2* overexpression (BioProject PRJNA523214) and mycorrhization (BioProject PRJNA509606) were submitted to the Panther tool. As shown in Table 1 and Supplementary Table 2, an overall overrepresentation of GO terms commonly associated with genes repressed by *SlDLK2* OE and induced by mycorrhization was observed, reflecting the negative regulatory role of *SlDLK2* on mycorrhization previously described by Ho-Plágaro et al. (2021). Among the *SlDLK2* OE-repressed and AM-induced gene sets, we found that many overrepresented GO terms are well-known to be activated during mycorrhization, such as the “cell wall modification,” “response to wounding,” “response to external stimulus,” “defense response,” and “transmembrane transport” GO terms from the biological processes category; the “transmembrane transporter activity,” “hydrolase activity,” “transcription regulatory activity,” and “antioxidant activity” GO terms from the molecular function category; and the “cell wall,” “cell periphery,” and “vacuole” GO terms from the cellular component category.

**Table 1:** Gene ontology of DEGs upon mycorrhization and *SlDLK2* overexpression for “Biological Process” category.

Biological process	Genes upregulated by SlDLK2 OE	Genes downreglated by SlDLK2 OE	Genes upregulated by mycorrhization	Genes downreglated by mycorrhization
Cellular lipid metabolic process		x	x	
Lipid metabolic process	x	x	x	
Isoprenoid catabolic process		x	x	
Response to chemical	x	x	x	
Response to stimulus		x	x	x
Response to inorganic substance		x		x
Cell wall modification		x	x	
Cell–cell junction assembly		x	x	
Cell junction assembly		x	x	
Cell junction organization		x	x	
Cell–cell junction organization		x	x	
Tyrosine catabolic process		x	x	
Tyrosine metabolic process		x	x	
Alpha-amino acid catabolic process		x	x	
Response to wounding		x	x	
Response to stress		x	x	x
Response to cytokinin		x	x	
Response to hormone	x	x	x	
Response to organic substance	x	x	x	
Response to endogenous stimulus	x	x	x	
Response to auxin		x	x	
Regulation of jasmonic acid mediated signaling pathway		x	x	
Biological regulation		x	x	
Regulation of defense response		x	x	
Regulation of response to stress		x	x	
Transport		x	x	
Establishment of localization		x	x	
Localization		x	x	
Transmembrane transport		x	x	
Inorganic cation transmembrane transport		x	x	
Inorganic ion transmembrane transport		x	x	
Fatty acid metabolic process		x	x	
Secondary metabolic process		x	x	
Negative regulation of catalytic activity		x	x	
Negative regulation of molecular function		x	x	
Homeostatic process		x	x	
Chemical homeostasis		x	x	
Defense response to other organism		x	x	
Response to other organism		x	x	
Biological process involved in interspecies interaction between organisms		x	x	
Response to external biotic stimulus		x	x	
Response to biotic stimulus		x	x	
Response to external stimulus		x	x	
Defense response		x	x	
Regulation of DNA-templated transcription		x	x	
Regulation of RNA biosynthetic process		x	x	
Regulation of RNA metabolic process		x	x	
Regulation of nucleobase-containing compound metabolic process		x	x	
Abscisic acid-activated signaling pathway	x		x	
Cellular response to abscisic acid stimulus	x		x	
Cellular response to alcohol	x		x	
Response to oxygen-containing compound	x			x
Cellular response to organic substance	x		x	
Cellular response to oxygen-containing compound	x		x	
Cellular response to hormone stimulus	x		x	
Cellular response to endogenous stimulus	x		x	
Cellular response to lipid	x		x	
Hormone-mediated signaling pathway	x		x	
Sterol metabolic process	x		x	
Carboxylic acid catabolic process	x		x	
Organic acid catabolic process	x		x	
Organic acid metabolic process	x		x	
Small molecule metabolic process	x		x	
Small molecule catabolic process	x		x	
Carboxylic acid metabolic process	x		x	
Oxoacid metabolic process	x		x	
Alpha-amino acid metabolic process	x		x	
Developmental process	x		x	

GO enrichment analysis for up- or downregulated genes (fold change >2 or < −2, respectively, and *p* < 0.05) in tomato roots upon mycorrhization or *SlDLK2* OE. Functional categories of biological processes commonly overrepresented (FDR <0.05) for the four gene sets are shown and labeled with a cross and a green (upregulated gene sets) or orange (downregulated gene sets) background. Hormone-related GO terms are indicated in red boxes. Analysis was performed using PhanterDB.

Hormone-related GO terms are indicated in red boxes.

Among the different GO terms commonly regulated by *SlDLK2* OE and mycorrhization, it caught our attention different GO terms associated with hormone regulation, response and signaling, such as “response to cytokinin,” “response to auxin,” “regulation of jasmonic acid mediated signaling pathway,” “abscisic-acid activated signaling pathway,” “isoprenoid catabolic process,” and “isoprenoid binding,” with fold enrichment values ranging from 2.5 to 9.8 (Tables 1, 2; Supplementary Table 2).

**Table 2:** Hormone-related gene ontology terms for DEGs upon *SlDLK2* overexpression.

	*SlDLK2* OE repressed genes		*SlDLK2* OE induced genes	
	Fold enrichment	FDR	Fold enrichment	FDR
Response to cytokinin	6.02	****		
Response to auxin	5.89	****		
Regulation of jasmonic acid mediated signaling pathway	5.89	***		
Isoprenoid catabolic process	4.34	***		
Response to hormone	2.58	****	3.59	***
Abscisic acid-activated signaling pathway (ABA receptors)			9.04	**
Cellular response to abscisic acid stimulus			9.04	**
Abscisic acid binding			9.80	**
Isoprenoid binding			9.04	**
Hormone binding			8.71	**

GO enriched terms related to hormones for *SlDLK2* OE up- and downregulated gene sets (fold change >2 or < −2, respectively, and *p* < 0.05) in tomato roots. Fold enrichment values and FDR are indicated (**, FDR < 0.01; ***, FDR < 0.001; ****, FDR < 0.0001). Analysis was performed using PhanterDB.

### 3.2 *SlDLK2* OE alters the expression of hormone-related genes

To identify hormonal pathways altered by *SlDLK2* OE, significantly induced or repressed genes (fold change >2 or < −2, respectively; *p*-value <0.05) upon *SlDLK2* overexpression (BioProject PRJNA523214) were submitted to the Plant MetGenMap tool. As shown in Table 3, the abscisic acid (ABA) and indoleacetic acid (IAA) biosynthesis pathways and the gibberellin inactivation pathway were significantly repressed, while the phaseic acid biosynthesis pathway, a process that is related to ABA degradation, was significantly induced. Regarding genes from the brassinosteroid biosynthesis pathway, the expression of both the *SlDLK2* OE-induced and *SlDLK2*-repressed gene sets significantly changed (Table 3).

**Table 3:** Predicted hormonal pathways altered by *SlDLK2* overexpression.

		*SlDLK2* OE-repressed genes	*SlDLK2* OE-induced genes
Hormone biosynthesis	Abscisic acid biosynthesis	**	
	Brassinosteroid biosynthesis II	*	*
	IAA biosynthesis I	*p* < 0.1	
Hormone degradation	Phaseic acid biosynthesis (related to ABA degradation)		**
	Gibberellin inactivation	*p* < 0.1	

Altered hormonal pathways upon *SlDLK2* OE predicted with the Plant MetGenMap tool based on the transcripts whose expression is significantly induced or repressed (fold change >2 or < −2, respectively; *p*-value <0.05) in the RNA-seq data from BioProject PRJNA523214 (*, *p* < 0.05; **, *p* < 0.01).

**Figure 1:**
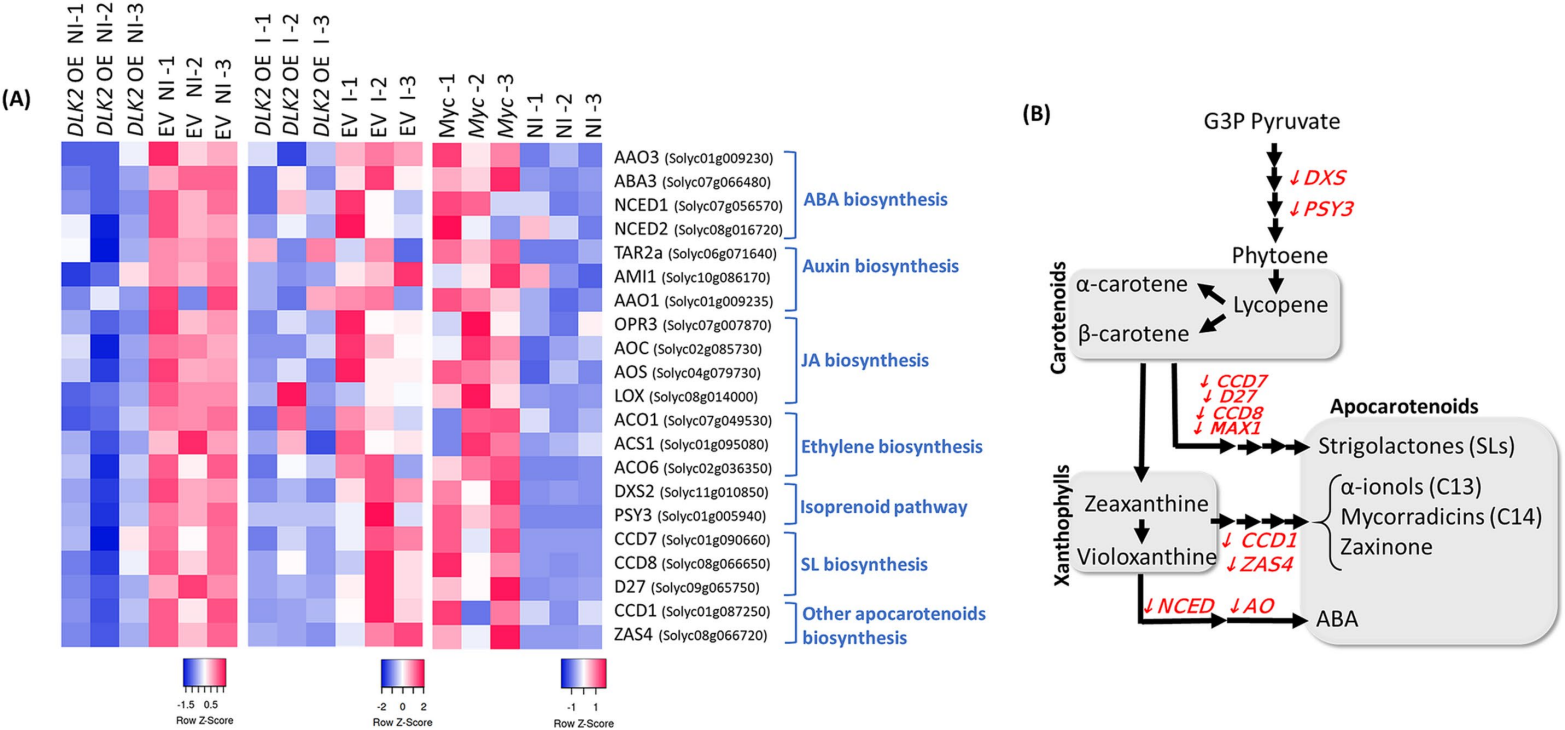
Repression of hormone-related genes by *SlDLK2* overexpression in roots. **(A)** Heatmaps of RNA-seq expression by computing the row *Z*-score using the normalized log2 count values for each gene. RNA-seq data correspond to *SlDLK2* overexpressing (*DLK2* OE) vs. control composite plants transformed with the empty vector (EV) (left heatmap; BioProject PRJNA523214), and mycorrhizal (Myc) vs. non-inoculated (NI) plants (right heatmap; BioProject PRJNA509606) (*n* = 3). **(B)** Schematic representation of the carotenoid and apocarotenoid biosynthesis pathway, indicating several genes involved with a significant repression in our RNA-seq analysis in the *SlDLK2* overexpressing roots with respect to the control (fold change<−2; *p*-value<0.05).

In a more detailed analysis, we pinpointed tomato genes that are putatively involved in different hormone pathways and we confirmed a clear repression of marker genes related to ABA and IAA biosynthesis, including the *AAO3*, *ABA3*, *NCED1*, *NCED2*, *TAR2a*, *AMI1,* and *AAO1* genes. Moreover, we found that *ACO1*, *ACO6,* and *ACS1* genes related to ethylene production, and *OPR3*, *AOC*, *AOS,* and *LOX* genes putatively involved in jasmonic acid biosynthesis were also repressed upon *SlDLK2* overexpression (left heatmap of Figure 1A). The repression of most of these hormone-related genes upon *SlDLK2* overexpression also occurred in mycorrhizal conditions (middle graph of Figure 1A), as shows the RNA-seq data from our experiment with *SlDLK2* OE composite plants and control plants transformed with the empty vector, both inoculated with the AM fungus *Rhizophagus irregularis* (Supplementary Tables 3, 4). More interestingly, a transcriptomic analysis comparing non-inoculated and mycorrhizal tomato roots (BioProject PRJNA509606) showed that the expressions of all these hormone biosynthesis genes that are repressed by *SlDLK2* are AM-induced genes (right heatmap of Figure 1A). In summary, our results show that *SlDLK2* overexpression triggers a negative regulation of a number of hormone biosynthesis genes, with a particular relevance of genes related to the biosynthesis of carotenoid and isoprenoid-derived plant hormones that are important molecules during mycorrhization such as apocarotenoids, gibberellins, ABA, *α*-ionols, or SLs, as illustrated in Figure 1B.

Moreover, a deeper analysis considering a wider number of putative tomato homologs of isoprenoid-related genes previously identified by Ezquerro et al. (2023) showed a repression of many genes related to the biosynthesis of other isoprenoid related molecules such as tocopherols and plastoquinone (Supplementary Table 5). Interestingly, the formation of monoterpenes, diterpenes, and tetraterpenes (carotenoids) and the prenyl moieties of chlorophyll, plastoquinone, and tocopherol requires the plastidial isopentenyl diphosphate (IPP) precursor (Pu et al., 2021), and indeed several genes from the methylerythritol phosphate (MEP) pathway responsible for the production of the plastidial IPP precursor are also repressed in *SlDLK2* OE roots. By contrast, the expression of genes from the mevalonate (MVA) pathway for the biosynthesis of cytosolic IPP pools which is related to the biosynthesis of other different compounds (sesquiterpene, triterpene, and polyterpene end products) was rather positively affected upon *SlDLK2* overexpression (Supplementary Table 5).

### 3.3 Isoprenoid biosynthesis genes and strigolactone contents are negatively affected in roots of *SlDLK2* OE stable-transformed tomato plants

**Figure 2:**
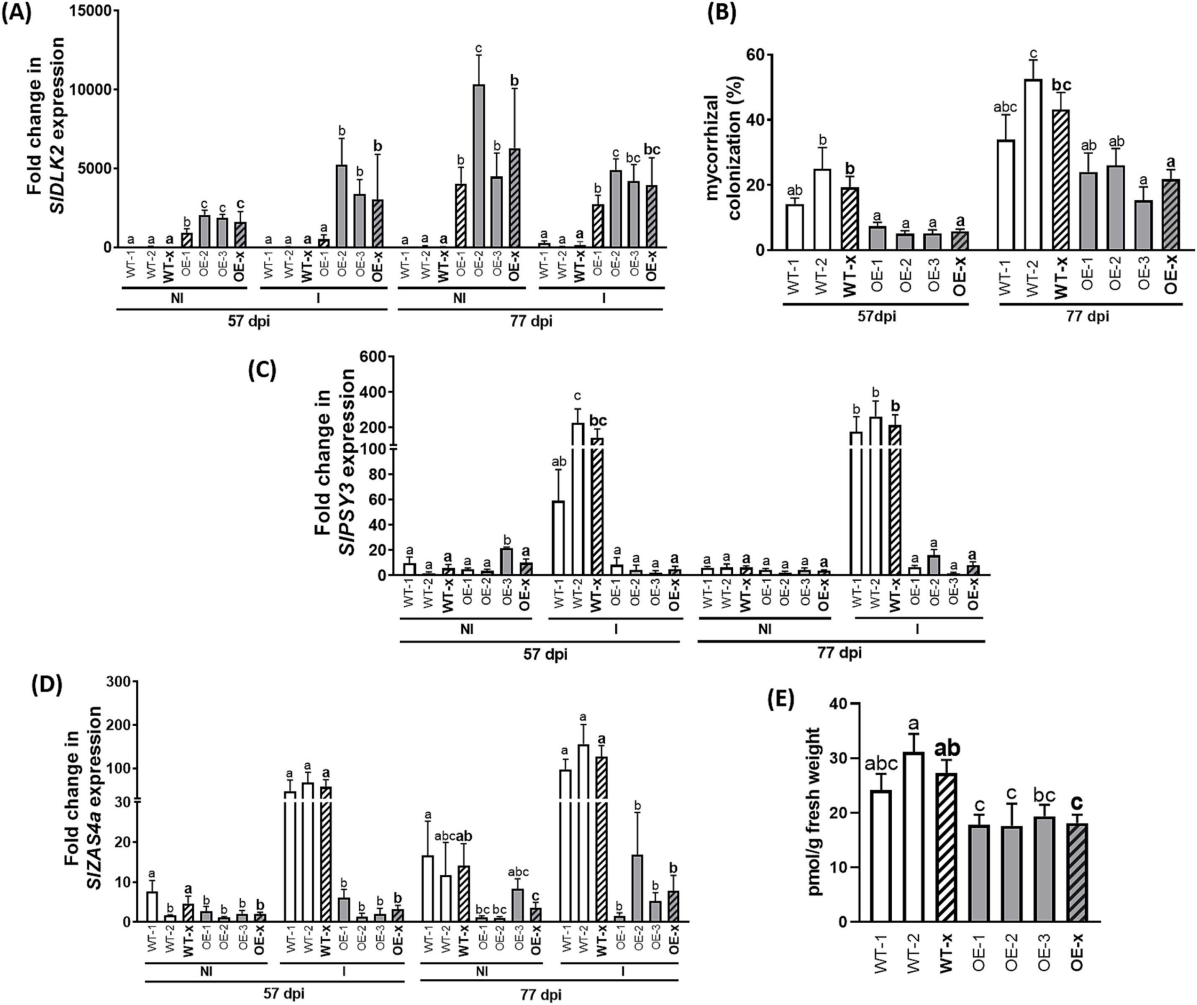
Expression of isoprenoid biosynthesis-related genes and strigolactone contents in roots from *SlDLK2* OE stable-transformed plants. A mycorrhizal experiment was performed with two transgenic-negative controls (WT-1 and WT-2) and three homozygous T3 *SlDLK2*-overexpressed tomato lines (OE-1, OE-2, and OE-3), and roots were analyzed after 57 and 77 days post-inoculation (dpi) with the AM fungus *Rhizophagus irregularis* (“I,” inoculated; “NI,” non-inoculated) (*n* > 6). **(A)** *SlDLK2* gene expression. **(B)** Percentage of total root length colonized by *R. irregularis* (*n* = 8). **(C,D)** Gene expression of the phytoene synthase 3 (*SlPSY3*) and zaxinone synthase (*SlZAS4*) genes, respectively. **(E)** Solanacol contents in root exudates of non-colonized control and *SlDLK2* OE lines under P-starvation conditions (*n* ≥ 3). qPCR data represent the relative gene expression with respect to the plant line showing the lowest expression, in which the corresponding gene expression was designated as 1. Striped bars correspond to the average of transgenic-negative controls (WT-x) and *SlDLK2* OE lines (OE-x). Values correspond to mean ± SE. Significant differences (Holm–Sidak’s multiple comparison test) are indicated with different letters (*p* < 0.05).

Stable tomato transgenic lines expressing p35S::*SlDLK2* were obtained using the pK7FWG2::*SlDLK2* binary vector and *Agrobacterium tumefaciens* LBA4404 for transformation. Two transgenic-negative controls (WT-1 and WT-2) and three homozygous T3 overexpressing lines (OE-1, OE-2, and OE-3) were selected on kanamycin-containing medium, and a mycorrhizal experiment with the AM fungus *Rhizophagus irregularis* was set up. We tested *SlDLK2* expression levels on the roots from these lines, in non-inoculated and mycorrhizal plants at 57 and 77 days post-inoculation (dpi). A successful overexpression of the *SlDLK2* gene was obtained in the *SlDLK2* OE plants in both conditions and at both harvesting points (Figure 2A).

First, we analyzed mycorrhizal colonization and transcriptional activity of isoprenoid biosynthesis-related genes. In agreement with the results obtained in our previous experiments with tomato composite plants overexpressing *SlDLK2* (Ho-Plágaro et al., 2021), a decreased mycorrhizal colonization was observed in the *SlDLK2* OE lines with respect to the control ones at 57 and 77 dpi (Figure 2B). Second, the expression of the phytoene synthase 3 (*SlPSY3*) and zaxinone synthase (*SlZAS4*) genes putatively related with isoprenoid biosynthesis during mycorrhization was induced by mycorrhization and repressed by *SlDLK2* OE in the absence of the AM fungi (Figures 2C,D), as expected based on the results of our previous RNA-seq analyses (Figure 1A). Moreover, we observed that *SlPSY3* and *SlZAS4* expressions were also reduced in the *SlDLK2* OE lines in mycorrhizal conditions at 57 and 77 dpi (Figures 2C,D), indicating that the negative effect of *SlDLK2* OE on isoprenoid biosynthesis genes is also occurring in AM plants.

To confirm whether strigolactone (SL) contents were reduced upon *SlDLK2* overexpression, we performed an experiment where *SlDLK2* OE and control plants were subjected to P-starvation conditions to promote SL production. As expected, SLs were not detected in the control treatment with P nutrition. By contrast, under P-starvation conditions, solanacol, which is one of the most abundant SLs in tomato (Kohlen et al., 2013), was detected, showing decreased levels in the *SlDLK2* OE roots (Figure 2E). These results confirm that *SlDLK2* overexpression causes a reduction of SL contents in roots.

### 3.4 Jasmonic acid, ABA, and indoleacetic acid contents are reduced in roots of *SlDLK2* OE stable-transformed tomato plants but not in leaves

**Figure 3:**
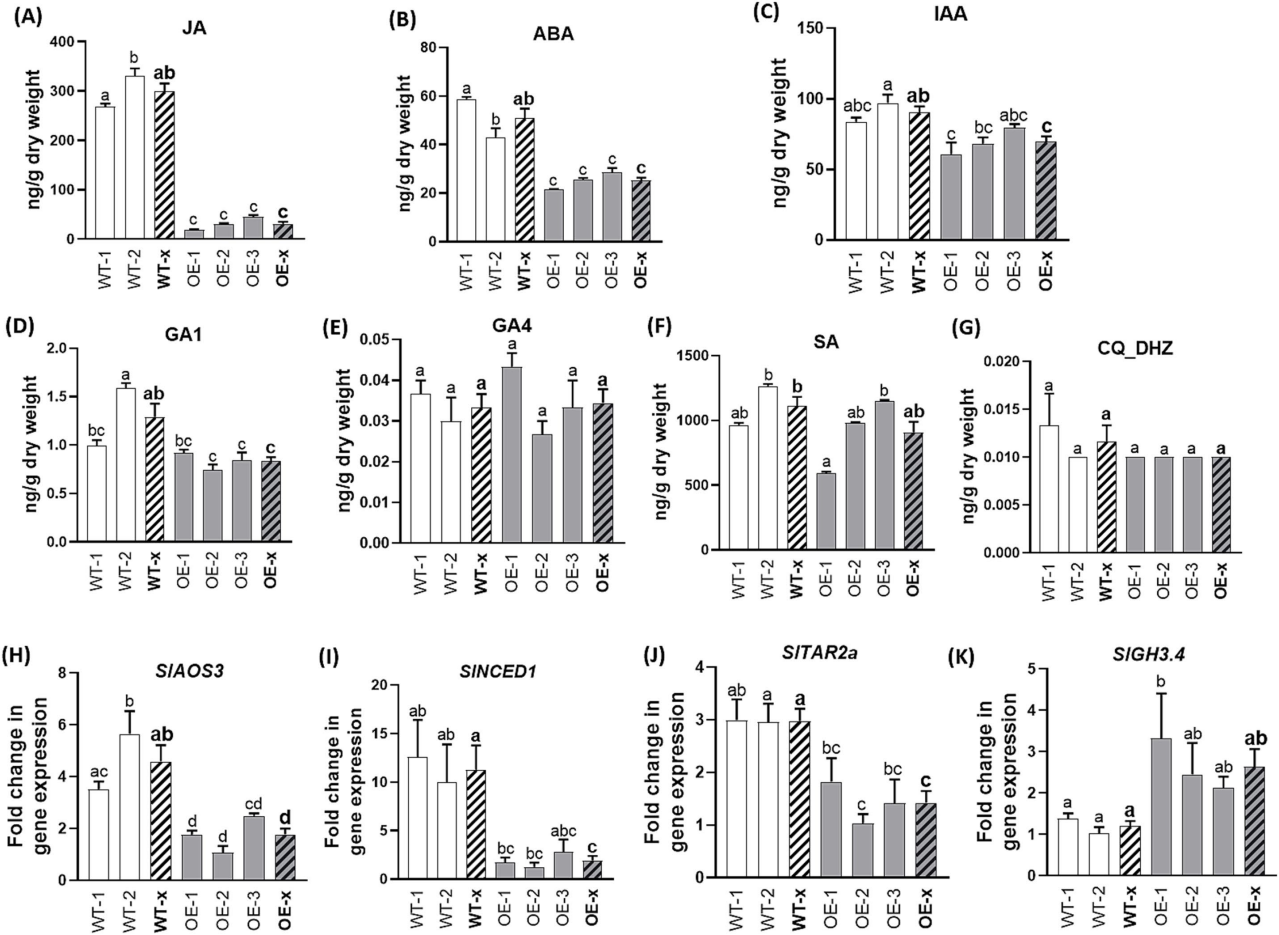
Hormone contents in roots of *SlDLK2* OE stable-transformed tomato plants. Hormone contents in roots of two transgenic-negative controls (WT-1 and WT-2) and three homozygous T3 *SlDLK2*-overexpressed tomato lines (OE-1, OE-2, and OE-3) grown for 57 days. **(A)** Jasmonic acid, JA; **(B)** abscisic acid, ABA; **(C)** indoleacetic acid, IAA; **(D)** gibberellin GA1; **(E)** gibberellin GA4; **(F)** salicylic acid, SA; **(G)** DHZ-type cytokinins, CQ DHZ. **(H–K)** Gene expression of the *AOS3* **(H)**, *NCED1* **(I)**, *TAR2a* **(J)**, and *GH3.4* **(K)** genes. qPCR data represent the relative gene expression with respect to the plant line showing the lowest expression, in which the corresponding gene expression was designated as 1. Striped bars correspond to the average of transgenic-negative controls (WT-x) and *SlDLK2* OE lines (OE-x). Values correspond to mean ± SE (*n* = 3). Significant differences (Holm–Sidak’s multiple comparison test) are indicated with different letters (*p* < 0.05).

To determine whether transcriptional changes in hormonal-related genes triggered by *SlDLK2* overexpression are accompanied by differences in hormonal contents, we selected non-mycorrhizal *SlDLK2* OE tomato lines grown for 57 days from the experiment explained above (Figures 2A–D). Mycorrhizal *SlDLK2* OE plants were not selected for hormonal content analysis to avoid possible effects due to the lower mycorrhization levels in these plants and not to a direct effect of *SlDLK2* overexpression. In the *SlDLK2* overexpressing roots, jasmonic acid content was strongly reduced (nearly a 10-fold decrease), and the ABA contents were significantly reduced to half with respect to the control tomato plants (Figures 3A,B). In addition, indoleacetic acid contents were slightly decreased in roots of *SlDLK2* OE plants, although this reduction was not significant for all tomato lines (Figure 3C). Not significant alterations were detected in the root content of gibberellins (GA1 and GA4), salicylic acid, and cytokinins (Figures 3D–G). As expected, the increase on the content of these hormones in *SlDLK2* OE roots was accompanied by a decreased expression of marker genes involved in the biosynthesis of JA (AOS3), ABA (*NCED1*), and IAA (*TAR2a*) (Itoh et al., 2002; Thompson et al., 2004; Mashiguchi et al., 2011), and an increased expression of the *GH3.4* gene related to auxin inactivation (Chen et al., 2022) (Figures 3H–K).

**Figure 4:**
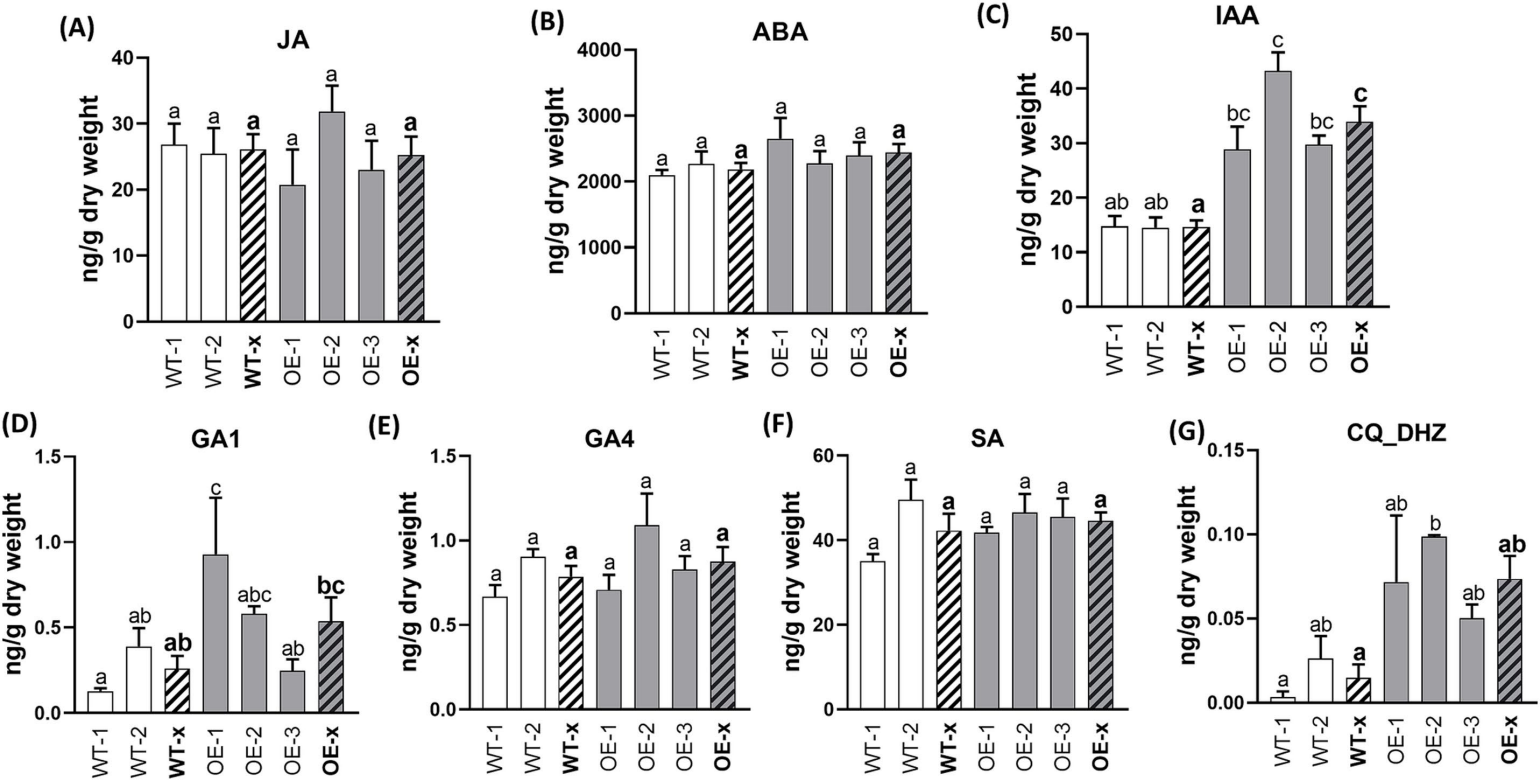
Hormone contents in leaves of *SlDLK2* OE stable-transformed tomato plants. Hormone contents in leaves of two transgenic-negative controls (WT-1 and WT-2) and three homozygous T3 *SlDLK2*-overexpressed tomato lines (OE-1, OE-2, and OE-3) grown for 57 days. **(A)** Jasmonic acid, JA; **(B)** abscisic acid, ABA; **(C)** indoleacetic acid, IAA; **(D)** gibberellin GA1; **(E)** gibberellin GA4; **(F)** salicylic acid, SA; **(G)** DHZ-type cytokinins, CQ DHZ. Striped bars correspond to the average of transgenic-negative controls (WT-x) and *SlDLK2* OE lines (OE-x). Values correspond to mean ± SE (*n* = 3). Significant differences (Holm–Sidak’s multiple comparison test) are indicated with different letters (*p* < 0.05).

Hormone content analysis was also performed on the leaves of the *SlDLK2* OE plants. In leaves, DHZ-type cytokinins showed a slight trend toward induction in the *SlDLK2* OE leaves, and indoleacetic acid was the only hormone found to be significantly affected by *SlDLK2* overexpression, showing increased levels in *SlDLK2* OE leaves with respect to the control leaves (Figure 4). Curiously, this result was opposite to the reduced levels of the indoleacetic acid observed in *SlDLK2* OE roots.

## 4 Discussion

D14 and KAI2 receptors that differentiate plant responses to SLs and KARs, respectively, belong to the RsbQ-like family of a,b-hydrolases. A third clade from this family is composed by the *DLK2* (DWARF 14-LIKE2) proteins, which are structurally similar to the D14/KAI2 receptors, but whose function is not so well-known. In tomato, SlDLK2 was recently shown to be involved in the complex plant-mediated signaling mechanism that regulates the life cycle of arbuscules and plays a central role in the negative regulation of arbuscule branching during AM formation (Ho-Plágaro et al., 2021).

Clear evidence shows that most phytohormones have an essential regulatory role from early stages in the presymbiotic signaling to later stages of AM development (revised by Pozo et al. (2015), Bedini et al. (2018), and Liao et al. (2018)). We observed that many gene ontology terms associated with hormone regulation, response, and signaling were commonly overrepresented upon *SlDLK2* OE and mycorrhization, suggesting that the role SlDLK2 on the regulation of mycorrhization might be mediated by a regulation of hormone balance. In this study, we show an in-depth analysis of the transcriptional changes triggered by *SlDLK2* overexpression in roots from composite plants based on the data obtained by Ho-Plágaro et al. (2021), and we focused our attention on a general repression of genes involved in hormone biosynthesis, with a special interest on the isoprenoid biosynthesis-related genes. In addition, we obtained stable-transformed *SlDLK2* OE lines, and we confirmed that the content of several hormones (JA, ABA, SLs, and probably auxins) was effectively reduced upon *SlDLK2* overexpression.

Gene expression and hormone content analyses revealed that several genes involved in jasmonic acid (JA) biosynthesis (*OPR3*, *AOC*, *AOS,* and *LOX*) were repressed in *SlDLK2* OE roots and that JA was the measured hormone showing the most strongly reduced contents (approximately a 10-fold decrease) upon *SlDLK2* overexpression (Figures 1, 3). Similarly, abscisic acid (ABA) and indoleacetic acid (IAA) contents were reduced in the *SlDLK2* OE roots (Figure 3), and this effect was accompanied by a repression of genes putatively involved in ABA (*AAO3*, *ABA3*, *NCED1*, and *NCED2*) and auxin (*TAR2a*, *AMI1*, and *AAO1*) biosynthesis (Figure 1), and an induction of genes putatively involved in ABA degradation (Table 3). Finally, strigolactone (SL) biosynthesis genes were also repressed (*CCD7*, *D27*, *CCD8,* and *MAX1*), and SL contents decreased in *SlDLK2* OE roots.

**Figure 5:**
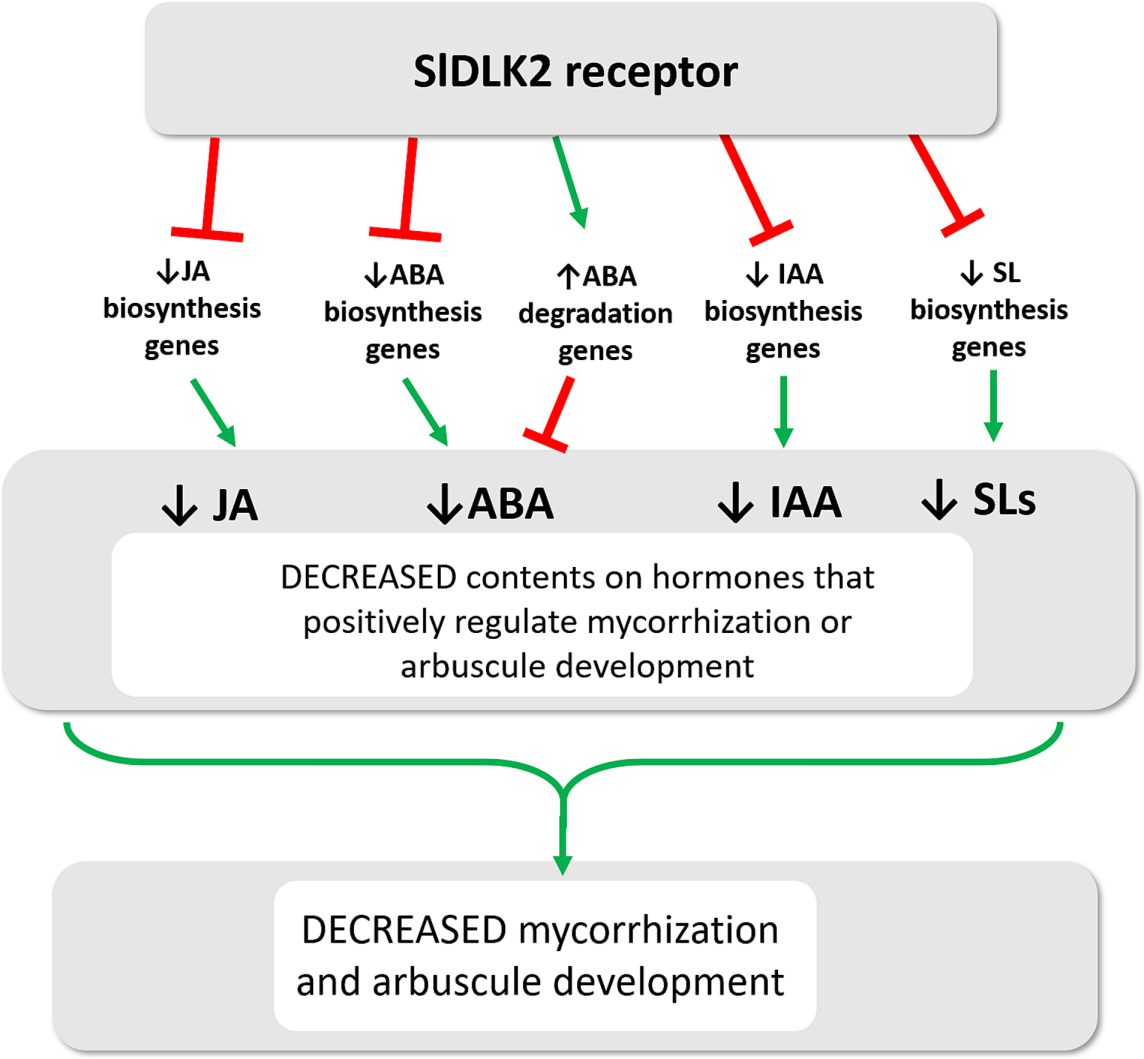
Model of SlDLK2-mediated signaling on hormone balance during AM symbiosis. *SlDLK2* gene induction occurs in arbuscule-hosting cells. The encoded SlDLK2 receptor represses a number of genes involved in the biosynthesis of several AM-promoting hormones including jasmonic acid (JA), ABA, indoleacetic acid (IAA), and strigolactones (SL), what triggers a negative effect on mycorrhizal colonization and arbuscule development.

Notably, for all these hormones (JA, ABA, SLs, and auxins) whose contents were reduced upon *SlDLK2* overexpression, a positive role on mycorrhization has been described. In the case of jasmonic acid (JA), although some conflicting data exist, multiple studies support its role as a positive regulator during mycorrhization (Herrera-Medina et al., 2008; Leon-Morcillo et al., 2012). For instance, tomato mutants deficient in JA (*spr2*) exhibit reduced mycorrhizal colonization, whereas the overexpression of prosystemin, which displays elevated JA levels, results in the opposite effect (Tejeda-Sartorius et al., 2008; Leon-Morcillo et al., 2012; Song et al., 2013; Casarrubias-Castillo et al., 2020). Abscisic acid (ABA), an apocarotenoid hormone, has been shown to significantly enhance mycorrhizal colonization and increase arbuscule intensity when applied exogenously, especially at low concentrations (Charpentier et al., 2014; Mercy et al., 2017). Conversely, tomato mutants with reduced ABA levels (*sitiens*) demonstrate lower mycorrhizal colonization and fewer well-developed arbuscules (Herrera-Medina et al., 2007; Martín-Rodríguez et al., 2011). Regarding strigolactones (SLs), another class of apocarotenoid phytohormones are critical for pre-symbiotic signaling. Under phosphate deficiency, SLs are secreted from plant roots into the rhizosphere, signaling the presence of a suitable host for colonization by arbuscular mycorrhizal (AM) fungi (Yoneyama et al., 2007; Kretzschmar et al., 2012). This signal stimulates fungal spore germination and hyphal growth, increasing the chances of physical contact between the fungus and host roots and preparing the fungus for symbiosis establishment (Akiyama and Hayashi, 2006; Besserer et al., 2006; Besserer et al., 2008; Kobae et al., 2018; Waters et al., 2017). SLs also induce fungal release of diffusible signals, such as short-chain chitin oligomers, which activate the common symbiosis signaling pathway (CSSP) in epidermal root cells, allowing initial colonization (Akiyama et al., 2005; MacLean et al., 2017; Waters et al., 2017). Consistent with these findings, mycorrhizal colonization is markedly reduced in plant mutants that are deficient in SL biosynthesis and transport (Koltai et al., 2010; Kretzschmar et al., 2012; Yoshida et al., 2012). Regarding auxins, several studies have also indicated that this hormone plays a role in the initiation of AM symbiosis as well as in the development and functionality of arbuscules (Hanlon and Coenen, 2011; Etemadi et al., 2014; Li et al., 2023). A positive correlation between endogenous indole-3-acetic acid (IAA) levels and the extent of mycorrhization, particularly arbuscule formation, has been demonstrated, suggesting that maintaining cellular auxin homeostasis is key to regulating AM symbiosis (Chen et al., 2022). Moreover, root auxin levels are associated with strigolactone exudation, and auxin may control early events in AM symbiosis by modulating SL levels (Foo, 2013). Finally, ethylene is mostly described as a negative regulator of AM in tomato (de Los Santos et al., 2011) and, although we found that genes related to ethylene biosynthesis were repressed upon *SlDLK2* overexpression, no changes in ethylene levels were observed in a preliminary analysis of ethylene exudation in control and *SlDLK2* OE plants (Supplementary Figure 1). In summary, experimental evidence highly supports the symbiotic positive role of all the hormones showing a reduction in the *SlDLK2* roots, suggesting that *SlDLK2* OE triggers a repression of JA, ABA, and auxin biosynthesis genes, what reduces the contents of these hormones in the roots and consequently contributes to negatively regulate mycorrhizal colonization, as illustrated in Figure 5.

Interestingly, a wide number of genes from the carotenoid/apocarotenoid pathway were repressed (Figure 1). Apocarotenoids are isoprenoid molecules produced in the plastids through the MEP pathway. In plants, the precursor of all isoprenoids is prenyl diphosphate (prenyl-PP), which is synthesized by two independent pathways: the mevalonate (MVA) pathway in the cytoplasm and the 2-C-methyl-d-erythritol 4-phosphate (MEP) pathway in plastids (Vranová et al., 2013). A number of studies show that the MEP pathway is induced during mycorradicin and is responsible for the production of many apocarotenoids that accumulate or are important during AM symbiosis, including not only ABA and strigolactones (SLs) but also other apocarotenoids such as C13 *α*-ionols, C14 mycorradicin, and zaxinone (Herrera-Medina et al., 2007; Yoneyama et al., 2007; Walter, 2020; Ablazov et al., 2023). In our study, we observed that the MEP pathway was repressed in roots overexpressing *SlDLK2*, suggesting that the lower mycorrhization upon *SlDLK2* overexpression might be due to the reduced biosynthesis of these AM signaling molecules that derive from the MEP pathway. By contrast, we observed that many genes putatively involved in the mevalonate pathway were induced in *SlDLK2* OE roots (Supplementary Table 5), probably as an indirect plant response to provide cytosolic IPP from the MVA pathway to the plastids to compensate the reduced IPP plastid precursors in these roots, as many studies show a crosstalk between cytosolic and plastidial IPP (Hemmerlin et al., 2003; Mendoza-Poudereux et al., 2015; Henry et al., 2018; Wagatsuma et al., 2018).

Supporting the strong induction of the MEP pathway upon *SlDLK2* overexpression, we observed that *SlDLK2* OE repressed the AM-inducible phytoene synthase *PSY3* gene (Figure 2). In *Medicago truncatula*, the PSY3 enzyme is known to be necessary for the production of strigolactones and C13 α-ionol/C14 mycorradicin apocarotenoids, and its knockdown negatively affects mycorrhization measured with the fungal marker *RiBTUB* (Stauder et al., 2018). Moreover, in agreement with our results, *PSY3* isogenes from tomato and *Medicago* are shown to be co-regulated with upstream genes (*DXS2*) and downstream carotenoid cleavage steps toward SLs (*D27, CCD7, and CCD8*), suggesting a coordinated induction of the carotenoid and apocarotenoid pathways for the delivery and usage of precursors for apocarotenoid formation, as proposed by Stauder et al. (2018). Moreover, we also observed that the AM-inducible gene encoding the zaxinone synthase *ZAS4* was also repressed in *SlDLK2* OE roots (Figure 2). The homolog genes *OsZAS* and *OsZAS2* from rice have an expression associated with arbusculated cells and are involved in the biosynthesis of AM-related apocarotenoids, being required for proper mycorrhizal colonization (Wang et al., 2019; Votta et al., 2022; Ablazov et al., 2023). Although OsZAS and OsZAS2 form zaxinone *in vitro*, an apocarotenoid that regulates strigolactone biosynthesis (Votta et al., 2022; Ablazov et al., 2023), contradictory results in experiments with exogenous zaxinone treatments suggest that in addition to zaxinone, these AM-related zaxinone synthases can form *in planta* a yet unidentified apocarotenoid for optimal mycorrhization, as proposed by Votta et al. (2022).

Finally, we investigated whether the hormonal alterations observed in roots of *SlDLK2* OE plants also occurred in leaves. The *DLK2* receptor is thought to have alternative potential functions in other tissues apart from its role in AM symbiosis, as *SlDLK2* is highly expressed in leaves and a photomorphogenic phenotype has been observed in *DLK2* mutants in the non-mycorrhizal plant *Arabidopsis* (Végh et al., 2017). In this study, we show that alterations in hormonal contents in the leaves of *SlDLK2* OE plants were not relevant (Figure 4), indicating that *SlDLK2* overexpression may participate in different specific signaling pathways in roots and leaves, probably by the coordinated action of *DLK2* with other elements that are specific of the different tissues.

Although overexpression can cause pleiotropic off-target effects by influencing multiple biological processes beyond the gene’s intended function, to date, ectopic gene expression is considered a valuable tool for gene functional characterization and for identifying candidate target genes through RNA-seq analyses. The role of SlDLK2 during mycorrhization has been previously described by Ho-Plágaro et al. (2021), using both RNAi and overexpression in composite plants with hairy roots, validating the use of this model for characterizing SlDLK2 functionality. Moreover, our RNA-seq results point to specific effects of *SlDLK2* overexpression, rather than off-target effects, as many genes from specific pathways are altered in the same direction. Reinforcing this idea, we observed that alterations in hormone content in leaves upon *SlDLK2* overexpression completely differ from those occurring in roots, where SlDLK2 is biologically active.

In summary, our study clearly shows that *SlDLK2* overexpression in the roots triggers an overall repression of genes for the biosynthesis of different hormones, with the consequent reduction in the levels of hormones with a previous described AM-promoting role, including jasmonic acid, auxins, and the apocarotenoid compounds ABA and strigolactones. Moreover, the general repression of genes from the MEP pathway and the apocarotenoid biosynthesis pathway indicates that the reduction in other apocarotenoid compounds might be also crucial for the regulatory function of *DLK2*. Altogether, we conclude that the *DLK2* receptor might be an important element for the repression of the biosynthesis of important hormones and apocarotenoids to negatively regulate mycorrhization.

To deepen the understanding of the underlying mechanisms, it would be highly interesting to perform protein–protein interaction assays between SlDLK2 and hormone-biosynthetic enzymes or hormone-related transcription factors to elucidate the mechanisms behind the SlDLK2-mediated regulation of hormonal biosynthesis pathways during mycorrhization. In this regard, the suppressor proteins JASMONATE ZIM DOMAIN PROTEIN (JAZ) and MYC2 are key components in the crosstalk between jasmonic acid (JA) and other plant hormones in plant growth and stress responses. Specifically, the molecular cascade involving the JAZ-MYC2-DELLA-PIF signaling module has been suggested to participate in the crosstalk between JA and GA signaling pathways (Yang et al., 2019). In addition, JAZ-MYC2 participates in the crosstalk between JA and ABA signaling pathways, influencing plant growth and defense responses (Chen et al., 2011). Interestingly, SlDLK2 interacts with DELLA (Ho-Plágaro et al., 2021), a protein that regulates arbuscule formation and degradation in AM roots. Accordingly, we can speculate that specific SlDLK2–DELLA interactions may interfere with both DELLA’s role as an activator of the transcription of plant genes required for arbuscule formation (Pimprikar et al., 2016), as well as the hormonal signaling crosstalk pathways in which DELLA is implicated. Furthermore, although we have proposed a direct suppression of JA and ABA biosynthesis by SlDLK2, the complex feedback loops intrinsic to hormone signaling pathways warrant further investigation. For instance, it is well-established that ABA can induce JA biosynthesis under certain conditions (Wang et al., 2018; Ju et al., 2019), suggesting that suppression of ABA could be expected to decrease JA levels as well.

## Data Availability

Publicly available datasets were analyzed in this study. This data can be found here: https://www.ncbi.nlm.nih.gov/bioproject/?term=PRJNA523214, https://www.ncbi.nlm.nih.gov/bioproject/?term=PRJNA509606.

## Footnotes

^1^ https://solgenomics.net/organism/Solanum_lycopersicum/genome

^2^ http://picard.sourceforge.net
