# Supplementary material for: The SlDLK2 receptor, involved in the control of arbuscular mycorrhizal symbiosis, regulates hormonal balance in roots
Source: Front Microbiol. 2024 Dec 11;15:1472449. doi: 10.3389/fmicb.2024.1472449 (PMC11668738; doi:10.3389/fmicb.2024.1472449)
Supplement: Supplementary file 1 [file Data_Sheet_1.DOCX]

Supplementary Material

# Supplementary Figures and Tables

## Supplementary Tables

**Supplementary Table 1.** Primers used in this study for quantitative reverse transcription polymerase chain reaction (RT-qPCR) experiments.

**Supplementary Table 2**. Gene ontology of DEGs upon mycorrhization and *SlDLK2* overexpression for “Molecular Function” and “Cellular Component” categories.

**Suplementary Table 3.** Number of mapped reads, high quality reads and splices reads for libraries from each sample in the RNA-seq analysis.

**Suplementary Table 4. List of genes detected in the RNA-seq analysis *SlDLK2* OE-I vs EV -I.** Fold change gene expression values and the p-values of *SlDLK2* OE roots from composite plants compared to control roots transformed with the empty vector (EV), both inoculated with the AM fungus *Rhizophaus irregularis* (Displayed as a separate excel file).

**Supplementary Table 5.** Response of isoprenoid related genes to mycorrhization or SlDLK2 overexpression.

## Supplementary Figures

**Supplementary Figure 1.** Ethylene production in roots of control and *SlDLK2* overexpressing tomato plants.

**Supplementary Table 1. Primers used in this study for quantitative reverse transcription polymerase chain reaction (RT-qPCR) experiments.**

| Target gene for qPCR  [SolDB accession number] | Primer name | Primer sequence (5´🡪3´) | Reference |
| --- | --- | --- | --- |
| ***SlEF-1α***  [Solyc06g005060.2] | qEF1α-F | (5´-GGTGGCGAGCATGATTTTGA-3´) | García Garrido et al., (2010) |
|  | qEF1α-R | (5´-CGAGCCAACCATGGAAAACAA-3´) |  |
| ***SlActin2***  [NM_001321306.1]  [Solyc11g005330.1] | qActin2-F | (5´- TTGCTGACCGTATGAGCAAG-3´) | Galpaz et al. (2006) |
|  | qActin2-R | (5´- GGACAATGGATGGACCAGAC-3´) |  |
| ***SlDLK2***  [Solyc05g018413] | qDLK2-F | (5´-GGGAGTTGAAATTGCATTACCT-3´) | García Garrido et al. (2010) |
|  | qDLK2-R | (5´-TAGTGAAATGGGCACCACAA-3´) |  |
| ***SlZAS4***  [Solyc08g066720] | qZAS4-F | (5´-ATGGAGGGCTTGCCAAATTTCA-3´) | [This](#_ENREF_1) work |
|  | qZAS4-R | (5´-CACAAGCCAACCATCATCCTCA-3´) |  |
| ***SlPSY3*** [Solyc01g005940] | qPSY3-F | (5´-AGCACTTCATTTGGGCATAGGG- 3´) | This work |
|  | qPSY3-R | (5´-GATCTGTCACTTTCCTCGCGAA-3´) |  |
| *SlAOS3*  [Solyc10g007960] | qAOS3-F | (5´- TCCAGATAGATTTGTGGGGG-3´) | García Garrido et al. (2010) |
|  | qAOS3-R | (5´- CCAATAAACCTTCCCATCAACA-3´) |  |
| *SlNCED1*  [Solyc07g056570] | qNCED2-F | (5´- CTTATTTGGCTATCGCTGAACC -3´) | Muñoz-Espinoza et al. (2015) |
|  | qNCED2-R | (5´- CCTCCAACTTCAAACTCATTGC -3´) |  |
| *SlTAR2a*  [Solyc06g071640] | qTAR2-F | (5´- CATCAATCTGGACCATGGTG -3´) | Alaguero‐Cordovilla et al. (2021) |
|  | qTAR2-R | (5´- TAGTCTGAGCATTACCAACTAG -3´) |  |
| ***SlGH3.4***  [Solyc02g092820] | qGH3.4-F | (5´-CTCCAGGGTGATTTCTGT-3´) | Liao et al. (2015) |
|  | qGH3.4-R | (5´-TTCTTTGGTCCACTGTCT-3´) |  |

**Supplementary Table 2. Gene ontology of DEGs upon mycorrhization and *SlDLK2* overexpression for “Molecular Function” and “Cellular Component” categories.** GO enrichment analysis for up or down-regulated genes (fold change >2 or <-2, respectively, and *P*<0.05) in tomato roots upon mycorrhization or *SlDLK2* OE. Functional categories of molecular functions and cellular components commonly over-represented (FDR <0.05) for the four gene-sets are shown and labelled with a cross and a green (for upregulated gene-sets) or orange (for downregulated gene-sets) background. Hormone-related GO terms are indicated in red boxes. Analysis was performed using PhanterDB.

**
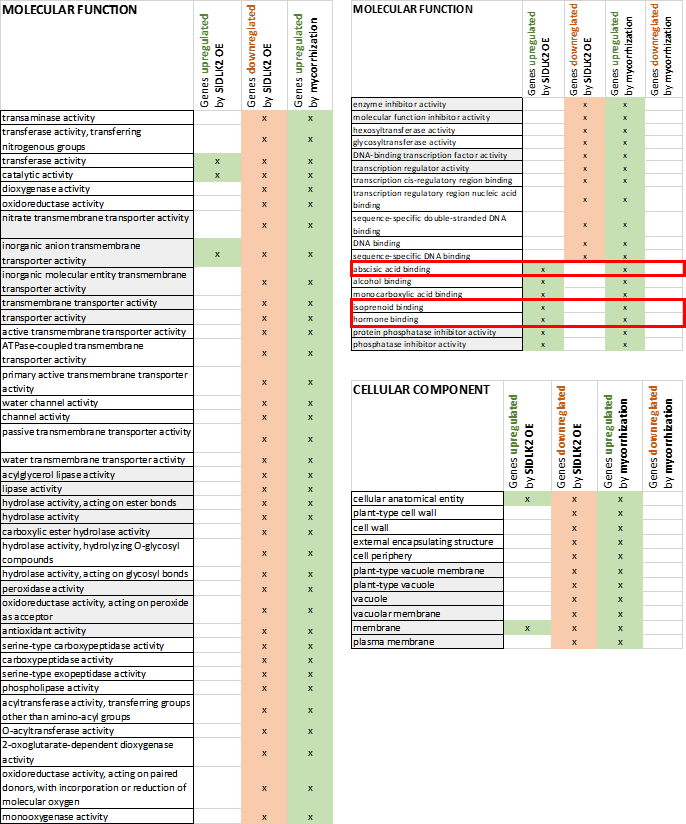
**

**Supplementary Table 3. Number of mapped reads, high quality reads and splices reads for libraries from each sample in the RNA-seq analysis.** Samples correspond to roots of composite plants inoculated with *R. irregularis* and transformed with the empty vector (EV I) or the SlDLK2 overexpression vector (SlDLK2 OE I).

|  |  | Mapped reads | | HQ reads | | Splice reads | |
| --- | --- | --- | --- | --- | --- | --- | --- |
| Sample name | Total reads | Number | % | Number | % | Number | % |
| EV I (1) | 80028166 | 68496728 | 85.59 | 59864742 | 74.8 | 17385711 | 21.72 |
| EV I (2) | 61858980 | 53304841 | 86.17 | 48202816 | 77.92 | 14240352 | 23.02 |
| EV I (3) | 53922282 | 49965276 | 92.66 | 38435124 | 71.28 | 11871351 | 22.02 |
| *SlDLK2* OE I (1) | 61326848 | 49580159 | 80.85 | 42485548 | 69.28 | 12084751 | 19.71 |
| *SlDLK2* OE I (2) | 61523798 | 54789629 | 89.05 | 46977534 | 76.36 | 12964421 | 21.07 |
| *SlDLK2* OE I (3) | 65171238 | 58418630 | 89.64 | 48021450 | 73.69 | 14875124 | 22.82 |
| Average  ± SE | 63.97 million ± 3.55 | 55.76 million ± 2.88 | 87.33 % ± 1.66 | 47.33 million ± 2.95 | 73.89 % ± 1.31 | 13.90 million ± 0.85 | 21.73 % ± 0.50 |

**Suplementary Table 4. List of genes detected in the RNA-seq analysis *SlDLK2* OE-I vs EV -I.** Fold change gene expression values and the p-values of *SlDLK2* OE roots from composite plants compared to control roots transformed with the empty vector (EV), both inoculated with the AM fungus *Rhizophaus irregularis* (Displayed as a separate excel file).

**Supplementary Table 5. Response of isoprenoid related genes to mycorrhization or SlDLK2 overexpression.** Putative tomato homologs of isoprenoid related genes were previously identified by Ezquerro et al. (2023) based on their homology to *Arabidopsis* genes. Fold change and p-values of these tomato genes is shown for two RNA-seq comparisons: inoculated with respect to non-inoculated plants ("I vs NI"), and composite plants overexpressing *SlDLK2* versus control plants transformed with the empty vector ("*DLK2* OE-NI vs EV-NI"). Significant up-regulation or down-regulation (fold change ≥2 or ≤-2, and P<0.05) are highlighted in green and red, respectively.

**
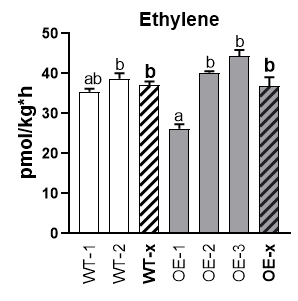
**

**Supplementary Figure 1. Ethylene production in roots of control and *SlDLK2* overexpressing tomato plants.** Ethylene content was measured in root systems from two transgenic-negative controls (WT-1 and WT-2) and three homozygous T3 *SlDLK2*-overexpressed tomato lines (OE-1, OE-2 and OE-3) grown for 57 days, according to Martín Rodríguez et al., (2011). Excised root systems were placed in a 16 mL test-tube sealed with a rubber stopper and incubated for 1 h at room temperature. The accumulation of ethylene in each tube was carried out in a Hewlett Packard 5890 gas chromatograph fitted with a flame ionization detector, using commercial ethylene as standard for identification and quantification purposes, and normalizing ethylene production to root mass. Striped bars correspond to the average of transgenic-negative controls (WT-x) and *SlDLK2* OE lines (OE-x). Values correspond to mean ± SE (n=5). Significant differences (Holm-Sidak´s multiple comparison test) are indicated with different letters (P < 0.05).

**References**

Alaguero‐Cordovilla, A., Sánchez‐García, A.B., Ibáñez, S., Albacete, A., Cano, A., Acosta, M., and Pérez‐Pérez, J.M. (2021). An auxin‐mediated regulatory framework for wound‐induced adventitious root formation in tomato shoot explants. *Plant, Cell & Environment* 44**,** 1642-1662.

Galpaz, N., Ronen, G., Khalfa, Z., Zamir, D. and Hirschberg, J. (2006) A chromoplast-specific carotenoid biosynthesis pathway is revealed by cloning of the tomato white-flower locus. *Plant Cell* 18: 1947-1960.

García Garrido, J.M., León Morcillo, R.J., Martín Rodríguez, J.A., and Ocampo Bote, J.A. (2010). Variations in the mycorrhization characteristics in roots of wild-type and ABA-deficient tomato are accompanied by specific transcriptomic alterations. *Molecular Plant-Microbe Interactions* 23**,** 651-664.

Liao, D., Chen, X., Chen, A., Wang, H., Liu, J., Liu, J., Gu, M., Sun, S., and Xu, G. (2015). The characterization of six auxin-induced tomato *GH3* genes uncovers a member, *SlGH3. 4*, strongly responsive to arbuscular mycorrhizal symbiosis. *Plant and Cell Physiology* 56**,** 674-687.

Martín‐Rodríguez, J.Á., León‐Morcillo, R., Vierheilig, H., Ocampo, J.A., Ludwig‐Müller, J., and García‐Garrido, J.M. (2011). Ethylene‐dependent/ethylene‐independent ABA regulation of tomato plants colonized by arbuscular mycorrhiza fungi. *New Phytol.* 190, 193-205.

Muñoz-Espinoza, V.A., López-Climent, M.F., Casaretto, J.A., and Gómez-Cadenas, A. (2015). Water stress responses of tomato mutants impaired in hormone biosynthesis reveal abscisic acid, jasmonic acid and salicylic acid interactions. *Frontiers in plant science* 6**,** 997.

**
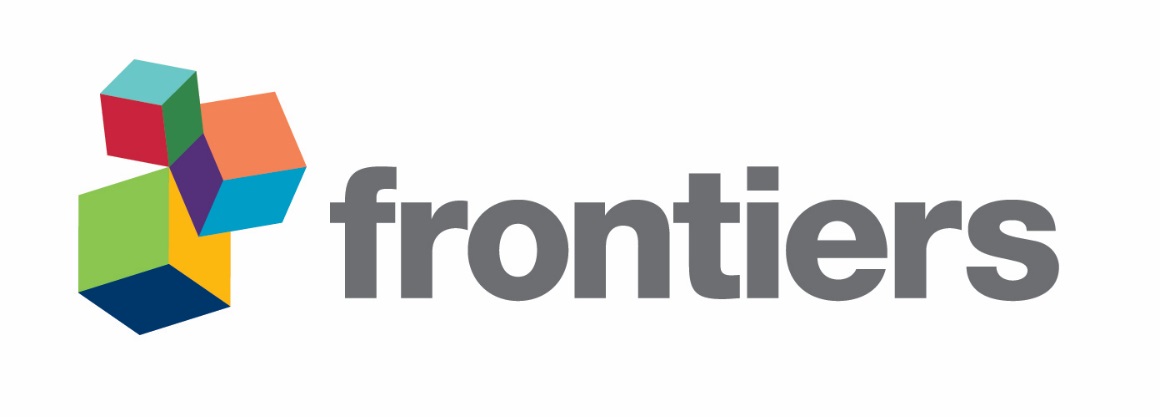
**
